# AZD1208, a pan‐Pim kinase inhibitor, inhibits adipogenesis and induces lipolysis in 3T3‐L1 adipocytes

**DOI:** 10.1111/jcmm.13559

**Published:** 2018-02-14

**Authors:** Yu‐Kyoung Park, Brice Wilfried Obiang‐Obounou, Kyung‐Bok Lee, Jong‐Soon Choi, Byeong‐Churl Jang

**Affiliations:** ^1^ Department of Molecular Medicine College of Medicine Keimyung University Daegu Korea; ^2^ Department of Food Science and Nutrition College of Natural Sciences Keimyung University Daegu Korea; ^3^ Division of Bioconvergence Analysis Korea Basic Science Institute Daejeon Korea; ^4^ Graduate School of Analytical Science and Technology Chungnam National University Daejeon Korea

**Keywords:** 3T3‐L1, adipogenesis, AMPK, AZD1208, lipolysis

## Abstract

The proviral integration moloney murine leukaemia virus (Pim) kinases, consisting of Pim‐1, Pim‐2 and Pim‐3, are involved in the control of cell growth, metabolism and differentiation. Pim kinases are emerging as important mediators of adipocyte differentiation. AZD1208 is a pan‐Pim kinase inhibitor and is known for its anti‐cancer activity. In this study, we investigated the effect of AZD1208 on adipogenesis and lipolysis in 3T3‐L1 cells, a murine preadipocyte cell line. AZD1208 markedly suppressed lipid accumulation and reduced triglyceride contents in differentiating 3T3‐L1 cells, suggesting the drug's anti‐adipogenic effect. On mechanistic levels, AZD1208 reduced not only the expressions of CCAAT/enhancer‐binding protein‐α (C/EBP‐α), peroxisome proliferator‐activated receptor‐γ (PPAR‐γ), fatty acid synthase (FAS), acetyl‐CoA carboxylase (ACC) and perilipin A but also the phosphorylation of signal transducer and activator of transcription‐3 (STAT‐3) in differentiating 3T3‐L1 cells. Remarkably, AZD1208 increased cAMP‐activated protein kinase (AMPK) and LKB‐1 phosphorylation while decreased intracellular ATP contents in differentiating 3T3‐L1 cells. Furthermore, in differentiated 3T3‐L1 adipocytes, AZD1208 also partially promoted lipolysis and enhanced the phosphorylation of hormone‐sensitive lipase (HSL), a key lipolytic enzyme, indicating the drug's HSL‐dependent lipolysis. In summary, the findings show that AZD1208 has anti‐adipogenic and lipolytic effects on 3T3‐L1 adipocytes. These effects are mediated by the expression and/or phosphorylation levels of C/EBP‐α, PPAR‐γ, FAS, ACC, perilipin A, STAT‐3, AMPK and HSL.

## 1 INTRODUCTION

Obesity is a major contributor to non‐communicable diseases, namely hyperlipidemia, type 2 diabetes and cancer.^1^ Its rise is attributed to many causatives, including nutritional and environmental factors, genetic and endocrine abnormalities, and imbalanced energy homeostasis.^2^ While adipose tissue is used as an energy reservoir, its role is critical in the control of energy metabolism when secreting adipokines.^2^, ^3^ Yet, recent evidence points to abnormal expansion of adipose tissue, closely linked to the development of obesity, is due to excessive adipocyte differentiation.^3^, ^4^

Adipocyte differentiation, also called adipogenesis, is the process during which fibroblast‐like preadipocytes develop into mature adipocytes.^5^ Adipocyte differentiation is controlled by the family of CCAAT/enhancer‐binding proteins (C/EBPs) and peroxisome proliferator‐activated receptors (PPARs)^6^, ^7^ and Janus‐activated protein kinase/signal transducer and activator of transcription (STAT) signalling complexes.^8^ In addition, the expression and/or activity of lipogenic enzymes, such as fatty acid synthase (FAS) and acetyl‐CoA carboxylase (ACC), and lipid droplet (LD)‐associated proteins, like perilipin A, are required for adipocyte differentiation.^9^, ^10^, ^11^, ^12^ A number of researchers reported about the involvement of cAMP‐activated protein kinase (AMPK), protein kinase A (PKA), extracellular signal‐regulated protein kinase‐1/2 (ERK‐1/2) and adenosine 3′,5′‐cyclic monophosphate (cAMP) in the control of adipocyte differentiation.^10^, ^13^, ^14^

Lipolysis is defined as the hydrolytic cleavage of ester bonds in triglyceride (TG), resulting in the generation of fatty acids and glycerol.^15^ Hormone‐sensitive lipase (HSL) is a key enzyme in the mobilization of fatty acids from stored TG^16^ and is phosphorylated (activated) by PKA, ERK‐1/2 and cAMP.^16^, ^17^ Thus, any compound that inhibits excessive adipogenesis and/or promotes lipolysis in adipocytes could be a therapeutic option against obesity.

The proviral integration site for moloney murine leukaemia virus (Pim) kinases, consisting of Pim‐1, Pim‐2 and Pim‐3, are active serine (S)/threonine (T) kinases that are constitutively expressed and are involved in the control of cell cycle, proliferation, and survival of normal and cancer cells.^18^ Due to the oncogenic/pro‐survival role in cancer biology, Pim kinases are recognized an interesting target for anti‐cancer therapy. However, recent evidence suggests Pim kinases as important mediators of adipocyte differentiation, with Pim‐2 considerably expressed in adipocytes,^19^ and the expression of Pim‐1 in adipocytic neoplasm as a marker of adipocytic differentiation.^20^ We have recently shown that a pan‐Pim kinase inhibitor SGI‐1776 blocks adipogenesis in differentiating 3T3‐L1 cells by reducing the expression and/or phosphorylation levels of C/EBP‐α, PPAR‐γ, FAS and STAT‐3.^21^ Thus, inhibition of Pim kinases could be a novel therapeutic path against obesity.

AZD1208 is a thiazolidine that is highly selective for all Pim isoforms^22^ and is known for its anti‐cancer activity.^23^, ^24^ As of now, neither the anti‐obesity effect nor the mode of action of AZD1208 in adipocytes is reported. This is the first study reporting the anti‐adipogenic and lipolytic effects of AZD1208 on 3T3‐L1 adipocytes through control of the expression and/or phosphorylation levels of C/EBP‐α, PPAR‐γ, FAS, ACC, perilipin A, STAT‐3, AMPK and HSL.

## 2 MATERIALS AND METHODS

### 2.1 Drugs and antibodies

AZD1208 was purchased from Selleckchem (Houston, TX, USA). Primary antibodies for anti‐C/EBP‐α, anti‐PPAR‐γ, anti‐phospho (p)‐STAT‐3 (Y705), anti‐STAT‐3, anti‐Pim‐1, anti‐Pim‐2, anti‐Pim‐3, anti‐p‐Bad (S112), anti‐Bad as well as secondary goat anti‐rabbit and goat antimouse IgG antibodies were purchased from Santa Cruz Biotechnology (Delaware, CA, USA). Primary antibodies specific for anti‐p‐AMPK (T172), anti‐AMPK, anti‐p‐ACC (S79), anti‐ACC, anti‐p‐LKB‐1 (S428), anti‐LKB‐1, S6, anti‐p‐HSL (S563) and anti‐p‐HSL (S660) were acquired from Cell Signaling Technology (Danvers, MA, USA). Anti‐HSL antibody was obtained from Cayman chemical (Ann Arbor, MI, USA). Anti‐perilipin A antibody was purchased from Bio Vision (Milpitas, CA, USA). Anti‐FAS antibody was purchased from BD Bioscience (San Jose, CA, USA). Anti‐actin antibody, 3‐isobutyl‐1‐methylxanthine (IBMX), dexamethasone and insulin were purchased from Sigma (St. Louis, MO, USA).

### 2.2 Cell culture and differentiation

3T3‐L1 preadipocytes (ATCC, Manassas, VA, USA) were grown up to the contact inhibition stage in DMEM supplemented with 10% heat‐inactivated foetal calf serum (FBS) (Gibco, Grand Island, NY, USA) and penicillin/streptomycin (Welgene, Daegu, Korea). Differentiation was induced by DMEM supplemented with 10% FBS plus a cocktail of hormones (MDI), containing 0.5 mmol L^−1^ IBMX (M), 0.5 μmol L^−1^ dexamethasone (D) and 5 μg/mL insulin (I) in the presence or absence of AZD1208. After 48 hours, the differentiation medium was replaced with DMEM supplemented with 10% FBS and 5 μg/mL insulin in the presence or absence of AZD1208 for 3 days. The cells were fed every other day with DMEM containing 10% FBS in the presence or absence of AZD1208 until day 8.

### 2.3 Oil red O staining

On day 8 of differentiation, the mock or AZD1208‐treated 3T3‐L1 cells were washed with PBS, fixed with 10% formaldehyde for 2 hour at room temperature (RT), washed with 60% isopropanol and dried. The fixed cells were stained with Oil Red O working solution (Sigma) for 1 hour at RT and washed with distilled water. LDs were observed using a light microscopy (Nikon, TS100, Japan).

### 2.4 Cell count analysis

3T3‐L1 preadipocytes seeded in 24‐well plates were similarly grown under the above‐mentioned differentiation conditions. On day 8 of differentiation, the mock or AZD1208‐treated 3T3‐L1 cells, which cannot be stained with trypan blue dye, was counted under microscope. The cell count assay was done in triplicates. Data are mean ± standard error (SE) of three independent experiments.

### 2.5 Quantification of intracellular TG contents by AdipoRed assay

On day 8 of differentiation, cellular lipid contents were measured with AdipoRed assay reagent kit in consonance with the manufacturer's instructions (Lonza, Basel, Switzerland). Fluorescence was measured after a 10 minutes incubation on Victor^3^ (PerkinElmer, Waltham, MA, USA) with excitation at 485 nm and emission at 572 nm.

### 2.6 Measurement of glycerol contents

Differentiated 3T3‐L1 adipocytes were serum‐starved for 2 hour and treated with AZD1208 or isoproterenol (ISO), a known lipolysis inducer, for 3 hour. Culture medium was saved and subjected to measure glycerol contents with a free glycerol reagent (Sigma) according to the manufacturer's instructions. The optical absorbance was determined at wavelength of 540 nm using the microplate reader.

### 2.7 Preparation of whole cell lysates

3T3‐L1 cells were washed with PBS and lysed in a modified RIPA buffer at a designated time point. The cell lysates were collected and centrifuged at 14 000 g for 15 minutes at 4°C. The supernatant was saved, and protein concentrations were determined with Pierce BCA Protein Assay Kit (Thermo scientific, Rockford, IL, USA).

### 2.8 Measurement of intracellular ATP levels

3T3‐L1 preadipocytes were seeded in 96‐well plates and grown in the presence of differentiation media in the absence or presence of AZD1208 or 2‐deoxyglucose (2‐DG), a glucose mimetic that depletes levels of cellular ATP. On day 2, 5 and 8 of differentiation, intracellular ATP levels were measured by luciferase activity using a luminescence assay kit according to the manufacturer's protocol (ATPLite‐1step, PerkinElmer). After 2 minute incubation, luminescence was measured on a Victor (PerkinElmer).

### 2.9 Western blot analysis

Proteins were separated by SDS‐PAGE and transferred onto nitrocellulose membranes (Millipore, Bedford, MA, USA). The membranes were washed with Tris‐buffered saline (10 mmol L^−1^ Tris‐Cl, 150 mmol L^−1^ NaCl, pH 7.5) supplemented with 0.05% (v/v) Tween 20 (TBST) followed by blocking with TBST containing 5% (w/v) non‐fat dried milk. The membranes were incubated overnight with specific primary antibodies at 4°C. The membranes were exposed to secondary antibodies conjugated to horseradish peroxidase for 2 hour at RT and treated with ECL reagents.

### 2.10 Quantitative real‐time RT‐PCR

Total cellular RNA in the mock or AZD1208‐treated 3T3‐L1 cells was isolated with the RNAiso Plus (Takara, Kusatsu, Shiga, Japan). Three micrograms of total RNA were reverse transcribed using a random hexadeoxynucleotide primer and reverse transcriptase. Single‐stranded cDNA was amplified by PCR with the following primers: C/EBP‐α sense 5′‐TTACAACAGGCCAGGTTTCC‐3′; antisense 5′‐GGCTGGCGACATACAGTACA‐3′; PPAR‐γ sense 5′‐AGGCCGAGAAGGAGAAGCTGTTG‐3′; antisense 5′‐TGGCCACCTCTTTGCTCTGCTC‐3′; FAS sense 5′‐TTGCTGGCACTACAGAATGC‐3′; antisense 5′‐AACAGCCTCAGAGCGACAAT‐3′; perilipin A sense 5′‐C ACTCTCTGGCCATGTGGA‐3′; antisense 5′‐AGAGGCTGCCAGGTTGTG‐3′; leptin sense 5′‐GACACCAAAACCCTCAT‐3′; antisense 5′‐CAGTGTCTGGTCCATCT‐3′; ACC sense 5′‐CAAGTGCTCAAGTTTGGCGC‐3′; antisense 5′‐CAAGAACCACCCCGAAGCTC‐3′; 18S rRNA sense 5′‐CCATCCAATCGGTAGTAGCG‐3′; antisense 5′‐GTAACCCGTTGAACCCCATT‐3′. SYBR Green PCR Master Mix (Takara) then analysed with the LightCycler^®^96 Machine (Roche, Mannheim, Germany). All reactions were performed in triplicate for each sample. Quantitation was performed by the comparative Ct (2eΔΔCt) method. The Ct value for each sample was normalized by the value for 18S rRNA.

### 2.11 Pim‐3 short‐hairpin RNA (shRNA) transfection

3T3‐L1 preadipocytes seeded into 6‐well plates were transfected for 6 hours with control or Pim‐3 shRNA using LipofectamineTM 2000 (Invitrogen, USA). Culture medium from the transfected cells was removed and refreshed with DMEM containing 10% FCS, followed by incubation for 48 hours. Differentiation of the control or Pim‐3 shRNA‐transfected 3T3‐L1 cells was induced as per the above‐mentioned differentiation conditions.

### 2.12 Statistical analysis

Cell count analysis was done in triplicates and repeated three times. Data were expressed as mean ± SE. The significance of difference was determined by one‐way ANOVA (Laerd Statistics, Chicago, IL, USA). All significance testing was based upon a *P* value of <.05.

## 3 RESULTS

### 3.1 AZD1208 inhibits lipid accumulation and reduces TG contents in differentiating 3T3‐L1 cells

**Figure 1:**
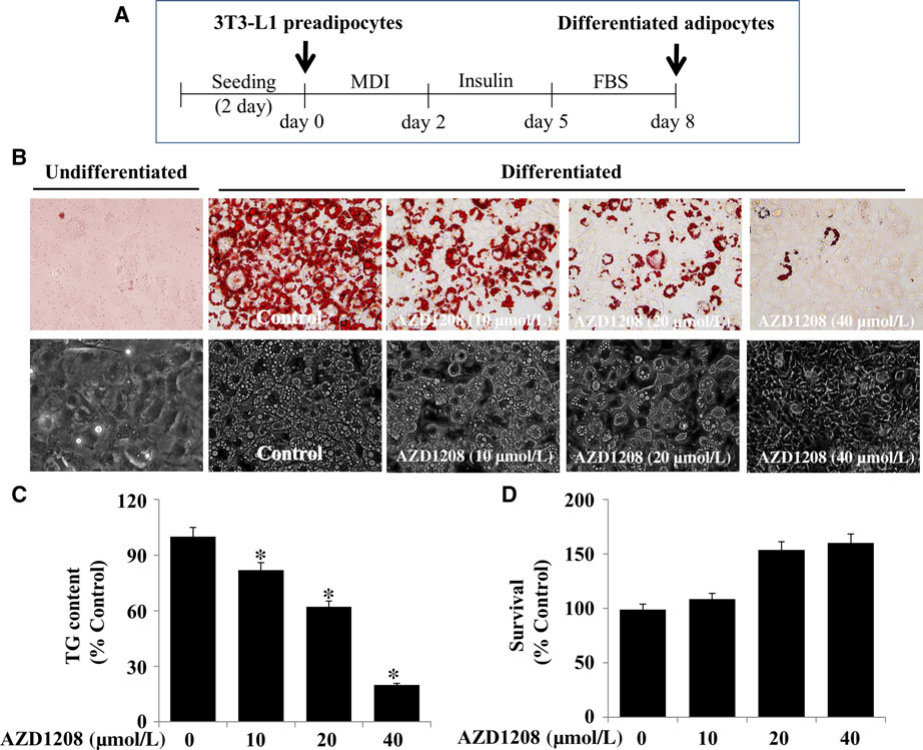
Effects of AZD1208 on adipogenesis and cell growth in differentiating 3T3‐L1 cells. A, The experimental scheme of 3T3‐L1 preadipocyte differentiation. B‐D, 3T3‐L1 preadipocytes were induced to differentiate with induction medium in the presence or absence of AZD1208 for 8 days. On day 8, cellular lipid contents were assessed by Oil Red O staining B, Phase‐contrast images of the cells were also taken after the treatment (lower panels in (B). On day 8, cellular TG contents were quantified by AdipoRed assay (C), respectively. Values are mean ± standard error (SE) of data from three independent experiments with three replicates. *P < .05 vs control. On day 8, AZD1208‐treated 3T3‐L1 cells, which cannot be stained with trypan blue dye, were counted under microscope (D). The cell count assay was done in triplicates. Data are mean ± SE of three independent experiments. *P < .05 vs control

To see the drug's anti‐adipogenic effect, we first determined whether AZD1208 inhibits lipid accumulation during the differentiation of 3T3‐L1 preadipocytes into adipocytes by an Oil Red O staining. The timescale of 3T3‐L1 preadipocyte differentiation is illustrated in Figure 1A. Many LDs were formed in differentiated 3T3‐L1 cells on day 8, as compared with undifferentiated cells (preadipocytes) (Figure 1B, upper panels). As compared with the mock‐treated cells, AZD1208 considerably decreased the amount of LDs in a concentration‐dependent manner. Evidently, treatment with 40 μmol L^−1^ AZD1208 strongly blocked the accumulation of LDs. The AZD1208's lipid‐reducing effect was also confirmed by light microscopy (Figure 1B, lower panels). We then examined whether AZD1208 reduces TG contents by an AdipoRed assay. AZD1208 also remarkably decreased the levels of TG in a dose‐dependent manner (Figure 1C). We finally looked at whether AZD1208 at the doses tested has cytotoxicity using cell count analysis. AZD1208 at the doses applied was not cytotoxic to 3T3‐L1 cells; rather, AZD1208 at 20 and 40 μmol L^−1^ enhanced the cell survival (Figure 1D). In consequence of strong inhibitory effects on lipid accumulation and TG synthesis with no cytotoxicity, the concentration of 40 μmol L^−1^ of AZD1208 was selected for more studies.

### 3.2 AZD1208 lowers the expression and/or phosphorylation levels of C/EBP‐α, PPAR‐γ and STAT‐3 in differentiating 3T3‐L1 cells

**Figure 2:**
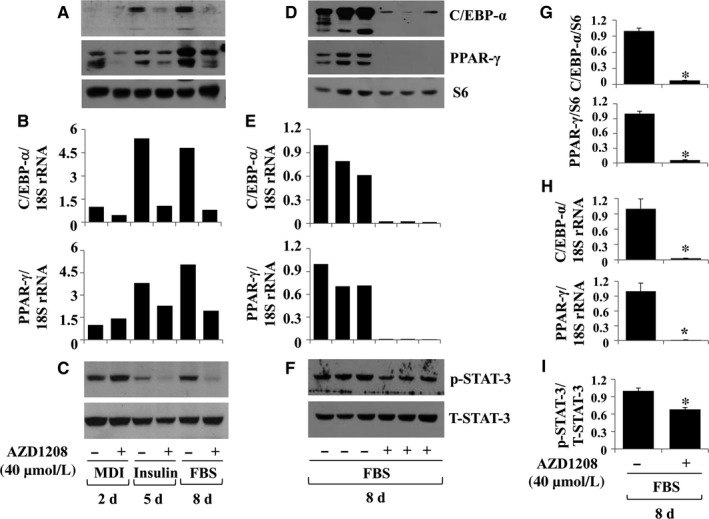
Effect of AZD1208 on expressions and/or phosphorylation of C/EBP‐α, PPAR‐γ and STAT‐3 in differentiating 3T3‐L1 cells. A‐C, 3T3‐L1 preadipocytes were induced to differentiate with induction medium in the presence or absence of AZD1208, and harvested at day 2, 5 and 8, respectively. Cellular protein and mRNA at the indicated time point were extracted and analysed by Western blot (A, C) and real‐time RT‐PCR (B) analysis, respectively. D‐F, Western blot (D, F) and real‐time RT‐PCR (E) analysis in triplicate experiments on day 8, respectively. G and H, the densitometry data of (D) and (E), respectively. I, The densitometry result of (F). *P < .05 compared to the value of AZD1208 free control on the indicated day

To understand the mechanisms of action of AZD1208‐mediated anti‐adipogenic effect, we examined whether AZD1208 affects the expression/activity (phosphorylation) of adipogenic transcription factors in differentiating 3T3‐L1 cells. AZD1208 inhibited the protein and mRNA expressions of C/EBP‐α and PPAR‐γ on days 2, 5 and/or 8 (Figure 2A,B). In addition, AZD1208 reduced STAT‐3 phosphorylation on days 5 and 8 (Figure 2C). The protein levels of STAT‐3 were not affected by the presence of AZD1208. Triplicate experiments confirmed the ability of AZD1208 to inhibit the expressions and phosphorylation of C/EBP‐α, PPAR‐γ and STAT‐3 on day 8 of differentiation (Figure 2D‐F). The densitometry data of Figure 2D,E,F are shown in Figure 2G,H,I, respectively.

### 3.3 AZD1208 down‐regulates the expressions of FAS, perilipin A and leptin in differentiating 3T3‐L1 cells

**Figure 3:**
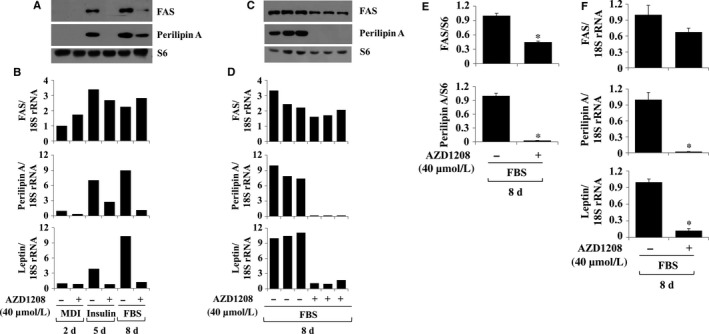
Effect of AZD1208 on protein and/or mRNA expressions of FAS, perilipin A and leptin in differentiating 3T3‐L1 cells. A, B, 3T3‐L1 preadipocytes were induced to differentiate with induction medium in the presence or absence of AZD1208, and harvested at day 2, 5 and 8, respectively. Cellular protein and mRNA at the indicated time point were extracted and analysed by Western blot (A) and real‐time RT‐PCR (B) analysis, respectively. C, D, Western blot (C) and real‐time RT‐PCR (D) analysis in triplicate experiments on day 8, respectively. E and F, the densitometry data of (C) and (D), respectively. *P < .05 compared to the value of AZD1208 free control on the indicated day

We next investigated the effect of AZD1208 on expressions of FAS and perilipin A in differentiating 3T3‐L1 cells. AZD1208 greatly suppressed protein levels of FAS and perilipin A on days 5 and 8 (Figure 3A). Moreover, AZD1208 reduced perilipin A, but not FAS, mRNA levels on days 2, 5 and 8 (Figure 3B). Adipose tissue is a major source of adipocytokines including leptin. Recent evidence suggests a role of leptin in obesity and related disorders.^25^ AZD1208 largely repressed leptinmRNA expression on days 2, 5 and 8. Triplicate experiments confirmed the ability of AZD1208 to inhibit the expressions of FAS, perilipin A and leptin on day 8 of differentiation (Figure 3C,D). The densitometry data of Figure 3C,D are shown in Figure 3E,F, respectively. Control S6 protein expression remained unchanged under these experimental conditions.

### 3.4 AZD1208 increases AMPK and LKB‐1 phosphorylation while decreases ACC phosphorylation in differentiating 3T3‐L1 cells

**Figure 4:**
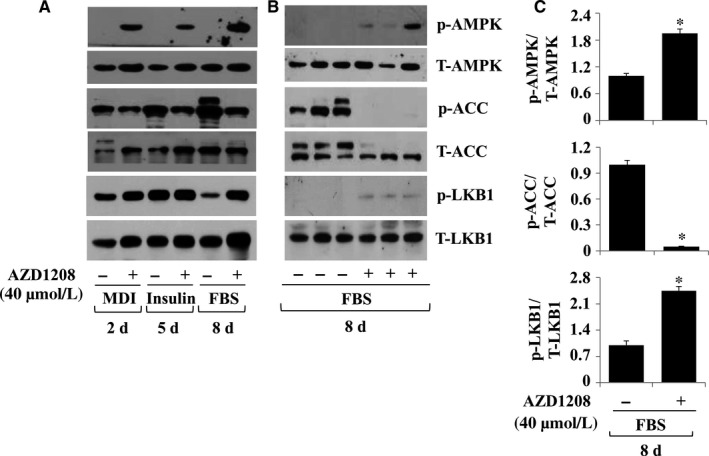
Effect of AZD1208 on expressions and phosphorylation of AMPK, ACC and LKB‐1 in differentiating 3T3‐L1 cells. A, 3T3‐L1 preadipocytes were induced to differentiate with induction medium in the presence or absence of AZD1208, and harvested at day 2, 5 and 8, respectively. Cellular protein at the indicated time point was extracted and analysed by Western blot analysis. B, Western blot analysis in triplicate experiments on day 8. C, the densitometry data of (B). *P < .05 compared to the value of AZD1208 free control on the indicated day

AMPK is a major regulator of energy metabolism, and its activation (phosphorylation) leads to inhibition of adipogenesis.^26^ We thus probed whether AZD1208 modulates AMPK phosphorylation in differentiating 3T3‐L1 cells. Notably, AZD1208 increased AMPK phosphorylation on days 2, 5 and 8 (Figure 4A). AZD1208 did not affect total AMPK protein levels. ACC is a best‐known downstream effector of AMPK and is involved in the biosynthesis of fatty acids.^27^ There was a time‐dependent increase in ACC phosphorylation during 3T3‐L1 preadipocyte differentiation. Yet, ACC phosphorylation was largely repressed by AZD1208 at the times tested. In addition, ACC total protein levels were reduced in the presence of AZD1208 on days 2 and 5, but not 8. Considering that liver kinase B‐1 (LKB‐1) is the main kinase to induce AMPK phosphorylation,^28^ we next measured whether LKB‐1 protein is expressed and activated (phosphorylated) in differentiating 3T3‐L1 cells and is regulated by AZD1208. In the absence of AZD1208, there were high levels of phosphorylated LKB‐1 on days 2 and 5 but largely declined on day 8 of differentiating 3T3‐L1 cells. Notably, on day 8 of differentiation, there were much elevated levels of LKB‐1 phosphorylation in AZD1208‐treated cells, compared with the mock‐treated ones. AZD1208 did not largely affect total protein levels of LKB‐1 on days 2, 5 and 8 of adipocyte differentiation. Triplicate experiments confirmed the ability of AZD1208 to increase AMPK and LKB‐1 phosphorylation but decrease ACC phosphorylation on day 8 of differentiation (Figure 4B). The densitometry data of Figure 4B are shown in Figure 4C.

### 3.5 AZD1208 decreases intracellular levels of ATP in differentiating 3T3‐L1 cells

**Figure 5:**
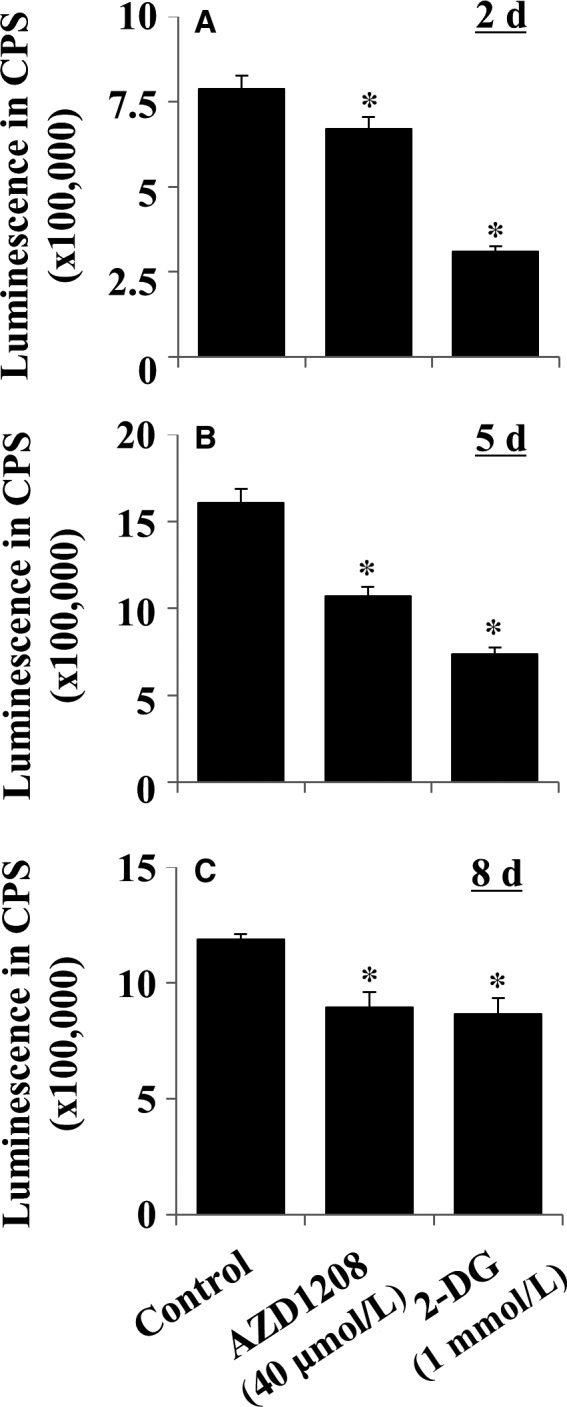
Effect of AZD1208 on intracellular levels of ATP in differentiating 3T3‐L1 cells. A‐C, 3T3‐L1 preadipocytes were induced to differentiate with induction medium in the absence or presence of AZD1208 or 2‐deoxyglucose (2‐DG), a known ATP depleting agent, and harvested at day 2 (A), 5 (B) and 8 (C), respectively. The amounts of intracellular ATP at the indicated time point were measured by an ATP measurement kit. *P < .05 compared to the value of AZD1208 or 2‐DG free control on the indicated day

AMPK phosphorylation is also induced by alterations in the intracellular AMP/ATP ratio.^29^ We thus examined whether AZD1208 affects intracellular ATP levels in differentiating 3T3‐L cells. In this study, 2‐deoxyglucose (2‐DG), a glucose mimetic that lowers intracellular ATP levels, was used as a control. As shown in Figure 5A‐C, 2‐DG substantially lowered cellular ATP contents on days 2, 5 and 8 of adipocyte differentiation. AZD1208 also had an ability to slightly reduce levels of cellular ATP on days 2, 5 and 8 of adipocyte differentiation.

### 3.6 AZD1208 enhances levels of Pim‐3 protein in differentiating 3T3‐L1 cells

**Figure 6:**
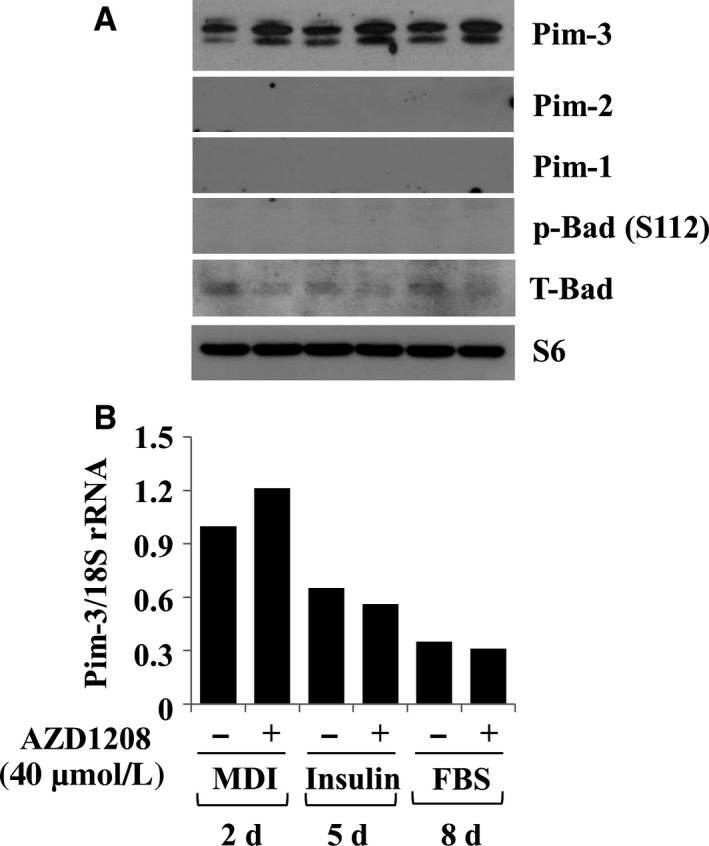
Effect of AZD1208 on expressions and/or phosphorylation of Pim kinases and Bad in differentiating 3T3‐L1 cells. A, B, 3T3‐L1 preadipocytes were induced to differentiate with induction medium in the presence or absence of AZD1208, and harvested at day 2, 5 and 8, respectively. Cellular protein and mRNA at the indicated time point were extracted and analysed by Western blot (A) and real‐time RT‐PCR (B) analysis, respectively. Each picture in (A) and (B) is a representative of three independent experiments

We next studied whether Pim kinases are expressed and AZD1208 alters their expression in differentiating 3T3‐L1 cells. Interestingly, substantial expression levels of Pim‐3, but not Pim‐2 and Pim‐1, protein were observed on days 2, 5 and 8 (Figure 6A). There was also substantial expression of Pim‐3 mRNA on days 2, 5 and 8 (Figure 6B). Strikingly, AZD1208 enhanced levels of Pim‐3 protein without affecting mRNA expression on days 2, 5 and 8. Pim kinases phosphorylate downstream effectors, such as Bcl‐2‐associated death promoter (Bad) protein on three serine (S) residues, including S112, S136 and S155.^30^, ^31^ We then looked at whether Bad is expressed and phosphorylated on S112, and AZD1208 affects it in differentiating 3T3‐L1 cells. Low levels of total Bad protein but no phosphorylated form of Bad were detected on days 2, 5 and 8. AZD1208 did not modulate Bad expression and phosphorylation; rather, the drug slightly reduced expression levels of total Bad on days 2, 5 and 8. Control S6 protein expression remained unchanged under these experimental conditions.

### 3.7 Knockdown of Pim‐3 does not affect lipid accumulation in differentiating 3T3‐L1 cells

**Figure 7:**
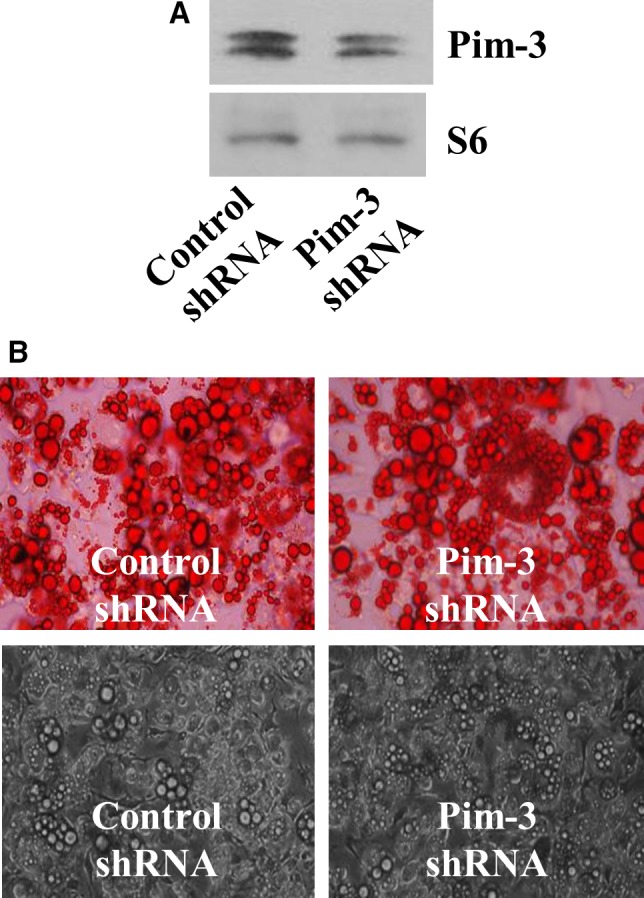
Effect of Pim‐3 shRNA transfection on lipid accumulation in differentiating 3T3‐L1 cells. A, B 3T3‐L1 preadipocytes were transfected with control shRNA or Pim‐3 shRNA for 48 h. The control or Pim‐3 shRNA‐transfected 3T3‐L1 preadipocytes were induced to differentiate with induction medium for 8 days. On day 8, cellular proteins were extracted and analysed by Western blot (A) and cellular lipid contents were measured by Oil Red O staining (B), respectively. Phase‐contrast images of the cells were also taken after the treatment (lower panels in B)

Using Pim‐3 shRNA transfection, we next studied the role of Pim‐3 in adipogenesis. Levels of Pim‐3 protein were substantially declined in Pim‐3 shRNA‐transfected 3T3‐L1 cells on day 8 of differentiation, as compared with control shRNA‐transfected cells (Figure 7A), suggesting the transfection efficiency. Control S6 protein expression remained unchanged under these experimental conditions. However, Pim‐3 knockdown did not influence lipid accumulation on day 8 (Figure 7B).

### 3.8 AZD1208 promotes lipolysis and HSL phosphorylation in differentiated 3T3‐L1 adipocytes

**Figure 8:**
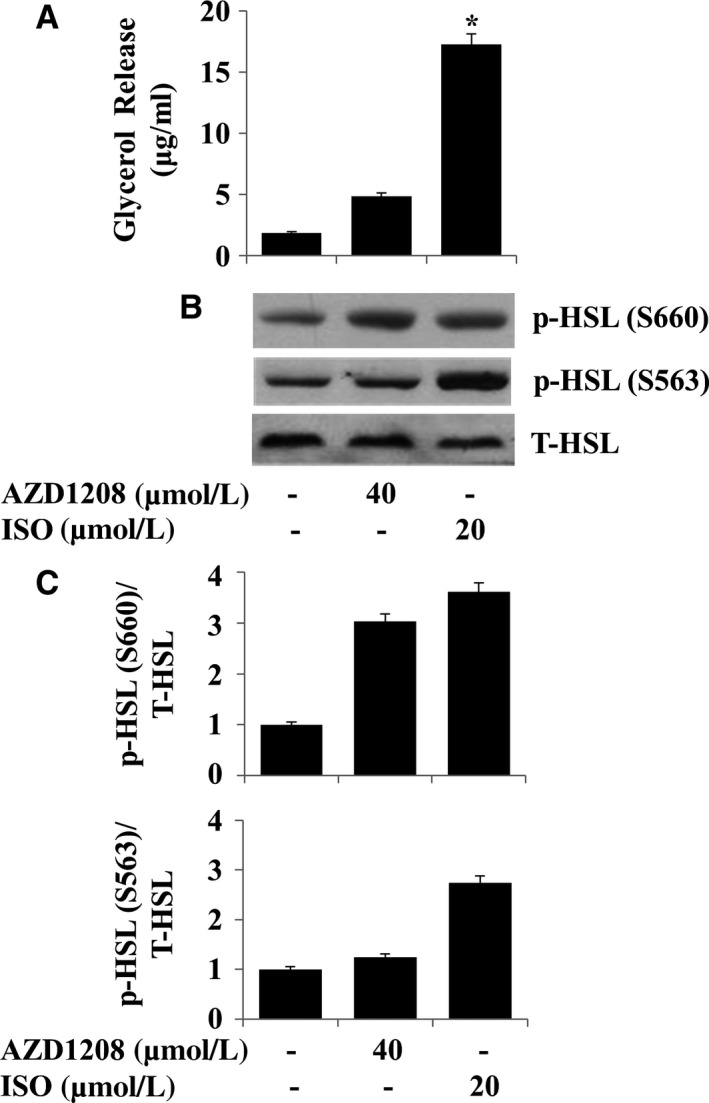
Effect of AZD1208 on lipolysis and HSL phosphorylation in differentiated 3T3‐L1 cells. A, Differentiated 3T3‐L1 cells were serum‐starved for 2 h and treated with AZD1208 or ISO for additional 3 h. Glycerol contents were measured in triplicates. Data are mean ± SE of three independent experiments. *P < .05 vs control. B, Differentiated 3T3‐L1 cells were serum‐starved for 2 h and treated with AZD1208 or ISO for additional 3 h. Cellular protein was extracted and analysed by Western blot analysis. Each picture in (B) is a representative of three independent experiments. C, The densitometry data of (B)

We next investigated the effect of AZD1208 on lipolysis in differentiated 3T3‐L1 adipocytes. The AZD1208's lipolytic effect was herein assessed by glycerol contents in the culture medium from the drug‐treated cells. Isoproterenol (ISO), a known lipolytic agent,^32^ was used as a positive control. In differentiated 3T3‐L1 adipocytes, ISO largely stimulated glycerol release while AZD1208 partly increased it (Figure 8A). Moreover, ISO strongly induced HSL phosphorylation on S563 and S660 (Figure 8B). AZD1208 greatly increased HSL phosphorylation on S660 but slightly enhanced HSL phosphorylation on S563. ISO or AZD1208 treatment did not largely affect total HSL protein level. The densitometry data of Figure 8B are shown in Figure 8C.

## 4 DISCUSSION

Pim kinases are known mediators of adipocyte differentiation. AZD1208 is a pan‐Pim kinase inhibitor and is known for its anti‐cancer activity. In order to reposition AZD1208 as an anti‐obesity alternative medicine, we investigated the drug's anti‐adipogenic and lipolytic effects on 3T3‐L1 adipocytes. Here, we demonstrate that AZD1208 inhibits adipogenesis and promotes lipolysis in 3T3‐L1 cells via control of the expression and/or phosphorylation levels of C/EBP‐α, PPAR‐γ, FAS, perilipin A, ACC, STAT‐3, AMPK and HSL.

AZD1208 strongly inhibits lipid accumulation and reduces TG contents in differentiating 3T3‐L1 cells, suggesting the anti‐adipogenic effect of the drug. Aforementioned, the expressions and activity of C/EBP‐α, PPAR‐γ and STAT‐3, are crucial for adipogenesis during adipocyte differentiation.^8^, ^33^, ^34^, ^35^ It is also documented that C/EBP‐α and PPAR‐γ are master transcriptional regulators of the entire terminal differentiation process. Considering that AZD1208 lowers the expressions of C/EBP‐α and PPAR‐γ and phosphorylation of STAT‐3 in differentiating 3T3‐L1 cells, the drug's anti‐adipogenic effect is largely due to down‐regulation of these adipogenic transcription factors. There is growing evidence that the expressions of adipocyte‐specific genes, such as FAS, perilipin A and adipokines, are required for adipocyte differentiation and maturation.^36^, ^37^ FAS is a lipogenic enzyme associated with the synthesis of fatty acid.^9^ During adipocyte differentiation, perilipin A binds to and stabilizes the newly formed LDs.^11^, ^12^ Because AZD1208 decreases cellular levels of FAS and perilipin A proteins in differentiating 3T3‐L1 cells, its anti‐lipogenic effect is through suppression of FAS protein expression. Furthermore, down‐regulation of perilipin A may further contribute to the drug's lipid‐lowering effect.

AMPK is a key regulator of energy metabolism and balance.^38^ It is a heterotrimeric protein kinase consisting of a catalytic α subunit and regulatory β and γ subunits. Recent evidence indicates that intracellular increase in the AMP/ATP ratio induces AMPK phosphorylation on T172 within the α subunit by liver kinase B1 (LKB1).^39^ A number of studies also have demonstrated that activation of AMPK inhibits ATP‐consuming anabolic processes while activates ATP‐producing catabolic processes,^40^, ^41^ and the effects are mediated via the phosphorylation of metabolic enzymes, such as ACC.^42^ There is also evidence that activation of AMPK induces ACC phosphorylation on S79, which strictly regulates the enzyme during fatty acid synthesis for malonyl‐CoA production, and phosphorylated ACC lacks its activity to synthesize fatty acids.^26^, ^43^ Interestingly, the Pim kinase‐mediated regulation of AMPK activity and energy metabolism has been previously proposed, with the facts that a novel Pim kinase inhibitor SMI‐4a induces activation of AMPK via increased AMP/ATP ratio in mouse embryonic fibroblasts deficient for all Pim isoforms.^44^ Given that AZD1208 increases AMPK phosphorylation but decreases ACC phosphorylation/expression in differentiating 3T3‐L1 cells, the drug's anti‐adipogenic effect is further mediated through AMPK activation and ACC down‐regulation, which may confer inhibition of ATP‐consuming anabolic processes, such as synthesis of fatty acids. Furthermore, in this study, AZD1208 increases LKB‐1 phosphorylation while lowers cellular ATP levels in differentiating 3T3‐L1 cells. These results indicate that there is no association between AMPK phosphorylation and ACC phosphorylation in AZD1208‐treated 3T3‐L1 cells, and the AZD1208‐induced AMPK phosphorylation is in part due to LKB‐1 activation and reduction of cellular ATP levels.

Our recent findings that a pan‐Pim kinase inhibitor SGI‐1776 suppresses adipogenesis^21^ and a meridianin C derivative, which has Pim kinase inhibitory activity,^45^ blocks adipogenesis^46^ suggest that inhibition of Pim kinases may provide a novel therapeutic approach to the treatment obesity. The notion is further supported by the AZD1208's anti‐adipogenic effect herein. Little is known about the expression and role of Pim kinases in adipogenesis. In this study, we show firstly that Pim‐3 protein is substantially expressed in differentiating 3T3‐L1 cells but knockdown of endogenous Pim‐3 does not affect lipid accumulation, revealing no role of Pim‐3 in adipogenesis. Expression of Pim kinases is regulated at multiple stages, including transcription, post‐transcription and translation. Because Pim kinases are constitutively expressed and active and have very short protein half‐life (<5 minutes), regulation of the protein stability is critical for their cellular function/activity. Remarkably, in differentiating 3T3‐L1 cells, AZD1208 enhances levels of Pim‐3 protein, but not mRNA, implying the drug‐mediated post‐transcriptional up‐regulation of Pim‐3. The present study reveals no detection (or change) of the phosphorylated Bad (S112) in differentiating 3T3‐L1 cells treated with AZD1208. Difficulty in detection of Bad phosphorylation/expression might be explained by very low or no expression of the phosphorylated form of Bad in the cells. To validate the drug efficacy, it will be necessary, in future, to analyse the phosphorylation and/or expression levels of other downstream components regulated by Pim kinases in AZD1208‐treated 3T3‐L1 cells. Pim kinases are expressed in normal and cancer cells and play a role in the cell growth/survival.^23^, ^24^, ^44^ Thus, assuming that knockdown of endogenous Pim‐3 does not interfere with lipid accumulation, AZD1208 largely inhibits adipogenesis, enhances Pim‐3 protein and increases cell survival in differentiating 3T3‐L1 cells on day 8, the drug's anti‐adipogenic effect is likely to be the Pim‐independent, and the up‐regulated Pim‐3 by AZD1208 may favour survival/growth of 3T3‐L1 cells at the late stage of differentiation.

In this study, we further show that AZD1208 has a lipolytic effect on differentiated 3T3‐L1 cells, as demonstrated by the drug's stimulating effects on glycerol release and HSL phosphorylation (S563, S660). Knowing that lipolysis occurs by activation of PKA which phosphorylates HSL on S563, S659 and S660 in adipocytes,^16^, ^17^ it is plausible that AZD1208 exerts its lipolytic effect via the PKA‐dependent HSL activation.

In summary, this is the first report demonstrating that AZD1208 has anti‐adipogenic and lipolytic effects on 3T3‐L1 adipocytes through control of the expression and/or phosphorylation levels of PPAR‐γ, C/EBP‐α, FAS, ACC, perilipin A, STAT‐3, AMPK and HSL. Even if important questions such as anti‐adipogenic and lipolytic effects of AZD1208 on obese animal models remain to be resolved, the present findings advocate AZD1208 as a potential therapeutics for the treatment of obesity.

## CONFLICT OF INTEREST

The authors declare no potential conflicts of interest.
